# Transcranial stimulation over the left inferior frontal gyrus increases false alarms in an associative memory task in older adults

**DOI:** 10.1097/01.hxr.0000491108.83234.85

**Published:** 2016-07-15

**Authors:** Ryan C. Leach, Matthew P. McCurdy, Michael C. Trumbo, Laura E. Matzen, Eric D. Leshikar

**Affiliations:** 1 University of Illinois at Chicago, Chicago, IL, USA; 2 University of New Mexico, Logan Hall, Albuquerque, NM, USA; 3 Cognitive Science and Applications, Sandia National Laboratory, Albuquerque, NM, USA

## Abstract

**Background:**

Transcranial direct current stimulation (tDCS) is a potential tool for alleviating various forms of cognitive decline, including memory loss, in older adults. However, past effects of tDCS on cognitive ability have been mixed. One important potential moderator of tDCS effects is the baseline level of cognitive performance.

**Methods:**

We tested the effects of tDCS on face-name associative memory in older adults, who suffer from performance deficits in this task relative to younger adults. Stimulation was applied to the left inferior prefrontal cortex during encoding of face-name pairs, and memory was assessed with both a recognition and recall task.

**Results:**

Face–name memory performance was decreased with the use of tDCS. This result was driven by increased false alarms when recognizing rearranged face–name pairs.

**Conclusions:**

This result suggests that tDCS can lead to increased false alarm rates in recognition memory, and that effects of tDCS on a specific cognitive task may depend upon cognitive capability for that task.

## Introduction

Older adults tend to suffer from cognitive deficits as a normal part of the aging process [1, 2]. An intervention called transcranial direct current stimulation (tDCS) can be used to enhance both motor and cognitive performance in older adults [3–5; for reviews, see 6–9]. TDCS works by sending a small electrical current through the scalp, which modulates neuronal activity. Anodal stimulation is thought to increase the likelihood of neuronal firing, and hence improve neural and cognitive function, whereas cathodal stimulation is thought to reduce the likelihood that neurons will fire an action potential, and thus inhibit neural function [8, 10]. Comparing active to sham stimulation, tDCS has been employed successfully in healthy older adult populations to improve performance on motor tasks [11], verbal fluency tasks [12], and working memory [13].

Older adults suffer specific deficits to episodic memory [14]. The associative memory deficit in older adults is the idea that they can better remember a given stimulus (i.e., item memory) than associations between stimuli (i.e., associative memory) [15]. Importantly, past work has demonstrated that tDCS can improve episodic memory performance in older adults, as measured by both recognition [16, 17], and recall tests [18, 19]. Proper name recall is improved with tDCS [20], and – important to the associative memory deficit – it has also improved object–location associative memory in older adults [21]. Taken together, this works suggests that use of tDCS may improve memory in older adults, but further work is needed to vet this possibility.

Not all tDCS studies, however, have successfully led to measurable improvements in performance, which constrains the claim that tDCS improves cognition. Although not all studies have focused exclusively on memory in older adults, there is a growing body of literature to suggest that tDCS does not universally improve cognition. For instance, aside from studies that have found no effects of tDCS on learning and memory performance [22, 23], application of tDCS has decreased performance in target detection [24], working memory [25,26], and general intelligence tasks [27]. Thus, it is important to study the delineation of conditions under which cognition improves because of stimulation rather than suffers from it.

How well participants perform on a given task at baseline may influence how effective tDCS is at improving performance. Some previous work suggests that tDCS does not improve performance in tasks when performance is low initially. For instance, one study found that tDCS improved performance on a working memory task in older adults, but only for those with a higher level of education [3], suggesting that the capability of tDCS to improve cognition depends on prior cognitive efficacy. Similarly, tDCS inhibited target detection only for those with low baseline performance, whereas high baseline performers were not affected by stimulation [24]. These results seem to indicate that the effects of tDCS on cognitive performance may differ with baseline cognitive performance.

Examining stimulation effects on face–name associative memory in older adults could be used to assess whether or not tDCS has an adverse effect on tasks where performance is low. Compared to younger adults, older adults have especially pronounced memory deficits in associative memory tasks where participants are asked to remember whether or not items were paired together [28, 29]. Indeed, older adults perform worse than younger adults on face–name associative tasks, even when memory for the names and faces themselves is relatively intact [30].

Our recent work has shown that tDCS improves this ability in younger adults [31], who do not show the same deficits in task performance. If, however, tDCS leads to decreased performance on tasks where performance is already low, it is possible that older adults’ performance might not increase, but that they might instead show decreased face–name memory. In other memory tasks, increasing false alarm rates has decreased performance with tDCS [32]. For this study, the authors deployed the Deese–Roediger–McDermott (DRM) task [33, 34] in which participants study category exemplars before the category theme is presented as a critical lure in a subsequent recognition task. Importantly, that work revealed that tDCS increased false alarms under active relative to sham stimulation. It is as yet unknown what effects tDCS might have on memory performance in healthy older adults on an associative memory task; a task in which older adults typically show low baseline performance and suffer from high rates of false alarms [15, 35].

In the present study, we tested the effects of tDCS on face–name associative memory in older adults. We predicted one of two possible outcomes: according to the benefit hypothesis, tDCS will benefit memory performance compared to sham activation, as it has in prior work [18]. According to the baseline hypothesis, however, tDCS will inhibit memory performance compared to sham, given that older adult performance on this task is low initially. Because some tDCS work has shown improved memory performance for older adults as measured by either recognition [17] or recall procedures [18], we chose to include both of these memory tests – few studies have used them simultaneously to further elucidate the effects of tDCS on memory (although some of our prior work presents an exception [31]).

For recognition, there are several possible effects of tDCS on memory. If tDCS improves memory in accordance with the benefit hypothesis, then stimulation may improve hit rates (i.e., judging that previously paired items, were in fact paired together), decrease false alarm rates (i.e., judging items that were not previously paired), or a combination of the two. However, if tDCS hurts memory performance on tasks where performance is low, then hit rates might decrease or remain unaffected under stimulation, but critically, false alarm rates would increase. In general, older adults tend to experience higher numbers of false alarms on associative tasks [35], so tDCS might reduce overall recognition performance by increasing false alarms. This possibility would be consistent with prior work [32].

Further, to assess whether tDCS may lead to poorer memory performance in a task where performance is low, we introduced within-subject manipulation to yield higher and lower memory performance levels to test whether performance changes are most pronounced because of changes in baseline performance.

## Methods

### Participants

Fourteen right-handed older adults (age range: 60–90 years) participated in this study, recruited from the Chicago surrounding community. Exclusion criteria were: not meeting the handedness criteria [36], and presence of pacemakers, cochlear or metal implants; history of skull fracture, brain injury or surgery; personal or familial history of epilepsy; cuts, scrapes, or abrasions to the scalp at the time of the experiment; all contraindications to tDCS. No participants showed signs of dementia or Alzheimer’s disease, as measured by the Mini Mental State Exam (MMSE [37]). All participants received $50 for their participation.

### Materials

Sixty faces from the FACES database [38], and 60 names taken from the Social Security Administration list of most common names (see [31]) were used as stimuli.

Faces depicted equal numbers of younger (aged 18–30 years), middle-aged (aged 39–55 years), and older adults (aged 69–80 years), as well as male and female targets.

Face stimuli were high quality images of people taken from the neck up in front of a gray background. Each face was assigned a name according to the Social Security Administration list of the most common names for that age group and gender. To counterbalance face–name pairs across participants, no name was paired with the same face across different versions of the experiment.

### Procedure

**Figure 1:**
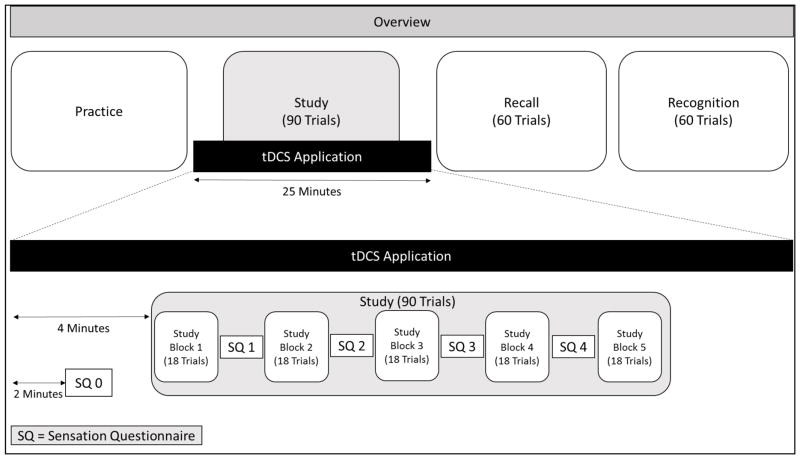
Overview of the study procedure. Participants practiced each session of the experiment before completing the stimulated study, recall, and recognition sessions. Stimulation began 4 minutes before the study session, and continued for 25 minutes. The recall session immediately followed stimulation cessation.

The three main phases of the experiment were the study phase (during which stimulation was applied), and the two test phases (recall and recognition; see Figure 1). After giving informed consent to participate in the study, subjects received instruction and training on each phase of the memory task during a practice phase. After attaching the electrodes and starting stimulation, participants were asked to sit quietly for 4 minutes to allow habituation to the sensations inherent to stimulation. Two minutes after stimulation began, participants filled out a Time 0 sensation questionnaire to rate their perception of sensation on a scale of 1 (very mild sensation) to 10 (extremely high, incredibly uncomfortable sensation) in terms of skin itching, burning, tingling, and mental fatigue. The study would be discontinued for any participants reporting a score of 7 or higher on any of these measures at any time, however this did not apply to any participant in our study. As stimulation continued, participants completed the study session, taking brief breaks to fill out further sensation questionnaires at four evenly spaced time points (Time 1–4). Specifically, participants completed blocks of 18 study trials, with a 1-minute break after each block (approximately every 2.5 minutes) to fill out the sensation questionnaire. At the end of each break, participants continued with the next study block, until all five study blocks were completed. Stimulation was discontinued after the study session; participants then completed the cued recall and recognition tasks, respectively. Participants then completed measures of fluid intelligence (digit symbol, and verbal fluency [39, 40]), crystallized intelligence (Shipley Vocabulary [41]), and the MMSE. Finally, participants filled out demographic and health questionnaires (Table 1).

### tDCS

Stimulation was administered to participants’ scalps via ActivaTek ActivaDose II controllers with saline-soaked square sponge electrodes (11 cm^2^). Two stimulators were used to administer an electric current of either 2.0 mA (active) or 0.1 mA (sham). Participants were assigned in a random and double-blinded manner to receive either active or sham stimulation, as done previously [31].

Blinding was executed by attaching both active and sham current generators to a blinding box with six settings, each connected to either the active or sham circuit unknown to the experimenter or the participant, as done in previous work [42]. Stimulation (active or sham) lasted exactly 25 minutes. The anodal electrode was placed on the scalp over the left inferior prefrontal cortex (located above F9 using the international 10–20 system and over the left sphenoid bone), and the reference electrode was placed on the contralateral upper arm. This brain region is known to be important in associative memory tasks in older adults [43, 44, 45].

### Study session

Participants studied 60 face–name pairs on a computer screen. Each trial was presented in a pseudo-random order for 8 seconds, split into two segments. First, participants were shown the face along with the name written in white, 18-point Arial font for 5 seconds. Second, the participants were given 3 seconds to indicate whether they thought the name “fit” the face, as has been done previously [45]. The purpose of this “fit” judgment was to orient attention to both the name and the face in order to facilitate associative memory. Trials were presented in blocks of approximately 2.5 minutes, and at the end of each block, participants rated their perceived physical sensations. As a manipulation of difficulty, half of the face–name pairs were presented twice and the rest only once. Thus, 90 trials were presented during study. After 25 minutes of stimulation, participants immediately began the memory test phases of the experiment.

### Cued recall test

During the self-paced cued recall task, participants viewed each of the 60 faces in a different pseudo-random order, with the proviso that no more than four faces from the same age group were presented in a row. For each face, participants were asked to type in the name associated with the face. Participants were instructed to type “no” if they did not remember the name.

### Recognition test

For the recognition task, participants were shown 60 face–name pairs. For half of trials, participants were shown the intact face–name pair they studied during encoding. For the other half of the trials, participants were shown rearranged pairs, whereby a face was presented with a different name to that presented at study. For the rearranged pairs, faces were only repaired with names matched for gender and age group (e.g., older female faces would only be re-paired with names associated with older female faces at study). For each recognition trial, participants judged whether each face–name pair was intact or rearranged in a self-paced manner. Recognition trials were presented in a new pseudo-random order with no more than four rearranged/intact trials presented in a row.

### Blinding probe

As a manipulation check of our blinding procedures, after the memory tests, participants were asked to indicate whether they thought they were in the active or sham condition, or if they could not tell the difference.

### Data analysis

To serve as a measure of recall performance, we calculated the proportion of names correctly typed by participants during the recall task (incorrect spellings were counted as correct). Several measures were used for recognition performance. Hit rate was calculated as the proportion of successfully recognized intact trials, and false alarm rates were calculated as the proportion of incorrectly indicated rearranged trials. Our corrected measure was A′, which takes into account both hit rate and false alarm rate [46]. Each of these four dependent variables (recall percentage correct, hit rate, false alarm rate, and A′) were used as dependent measures in the study.

## Results

### Behavioral measures

We were primarily interested in the effects of tDCS on memory performance, as measured by both recall and recognition tasks. Recall performance, hit rates, false alarm rates, and A′ scores are shown as a function of stimulation condition (active versus sham) and presentation (1 versus 2) in Table 2. Each measure was entered into a 2 (stimulation: active versus sham) × 2 (presentation: 1 versus 2) mixed ANOVA to test for tDCS effects, the effects of the difficulty manipulation (i.e., items presented once versus twice), and their interaction. For recall, only the main effect of difficulty manipulation was significant (*F*[1, 12] = 13.20, *p* < 0.05), showing that recall was significantly improved when participants studied trials twice rather than once.

There was, however, a marginal main effect of stimulation, such that participants in the sham condition outperformed those in the active condition in the recall of names (*F*[1, 12] = 3.54, *p* = 0.08).

For hit rate, only the main effect of difficulty manipulation was significant (*F*[1, 12] = 6.19, *p* < 0.05), showing that participants made more hits on trials presented twice than once. For false alarm rate, however, only the main effect of stimulation was significant (*F*[1, 12] = 8.37, *p* < 0.05), which indicated that false alarm rates were higher in the active condition than the sham condition. Finally, for A′ scores, both the main effects of difficulty manipulation (*F*[1, 12] = 6.69, *p* < 0.05) and stimulation (*F*[1, 12] = 7.39, *p* < 0.05) were significant. Participants in the sham condition outperformed those in the active condition, and participants performed better for trials presented twice versus only once. No interactions between difficulty manipulation and stimulation were significant for any of our memory measures (all *F*’s < 2.39).

Assessment of our blinding procedures revealed that, after the memory tests: 57% of participants could not tell which group they were in, 21% correctly guessed their condition, 14% answered incorrectly, and 7% did not indicate a response because of experimenter error. This suggests most participants could not tell which condition they were placed in.

### Sensation questionnaire

The means of physical sensations reported at each time point, split by stimulation condition, are reported in Table 3. Participants in the active condition provided significantly higher ratings than those in the sham condition at Time 0 for itching (*t*[12] = 3.25, *p* < 0.05), and burning (*t*[12] = 2.50, *p* < 0.05). At Time 1, only the differences in burning were significant (*t*[12] = 2.70, *p* < 0.05; itching *p* = 0.1), and all differences were marginal for both itching and burning at all time points thereafter (0.17 > all *p*’s > 0.05). These results suggest that participants initially felt significantly more itching and burning in the active condition than in the sham condition, and that these differences dissipated as the experiment progressed. To test whether these differences impacted memory performance, measures of itching and burning at each time point were entered into correlational analyses, with hit rates and false alarm rates as criterion variables. One marginally significant correlation was found between itching at Time 0 and false alarm rates (*r*[14] = 0.53, *p* = 0.053), suggesting that participants in the active condition may have been distracted by higher initial itching sensations than those in the sham condition. To test for overall differences in reported physical sensations across the entire stimulation period, time points of each physical sensation were combined into one measure. Participants reported more overall itching in the active condition (*M* = 2.74, *SD* = 1.59) than in the sham condition (*M* = 1.23, *SD* = 0.60; *t*[12] = 2.34, *p* < 0.05), and more overall burning in the active (*M* = 2.65, *SD* = 1.46) than sham condition (*M* = 1.23, *SD* = 0.41; *t*[12 = 2.49, *p* < 0.05), whereas there were no differences in tingling or mental fatigue (all *p*’s > 0.15).

## Discussion

The goal of this study was to examine face–name associative memory performance in older adults under tDCS. There were two key findings. First, active tDCS decreased recognition performance in older adults, primarily indexed by increased false alarm rates. Second, self-reported itching sensations were marginally correlated with higher rates of false alarms, suggesting that the sensory effects of stimulation may have interfered with memory processes.

Our results lend support to the baseline hypothesis of tDCS effects on memory, i.e., face–name associative memory performance was decreased in older adults compared to sham stimulation. This is contrary to what we have previously found in younger adults [31]. Because older adults suffer deficits for face–name associative memory at baseline compared to younger adults [30], our results suggest that tDCS effects on cognition may be moderated by differences in baseline performance; specifically tDCS may hinder performance but only in conditions of already poor performance. Our finding that tDCS led to decreased memory performance in a task where performance is typically low may explain why tDCS effects on cognition have been mixed in the past [47, 48]. Using anodal stimulation, some studies have found null effects on cognition [22, 23], whereas others have shown decreased cognitive performance [24, 27]. The moderating influences of baseline performance may influence when tDCS improves cognition, such as when only adults with a high level education show benefits in working memory performance [3].

We did not replicate our prior work in younger adults, which showed that active tDCS improved recall memory for face–name associations [31]. Instead, we found that in older adults, recognition memory decreased under stimulation on a task in which older adults typically perform poorly [29]; this supports the baseline hypothesis. Our finding of increased false alarms, however, is consistent with Pergolizzi and Chua [32], who found increased false memory rates under tDCS. This suggests that under certain conditions, such as with critical lures in the DRM paradigm, or in tasks where participants are more likely to falsely recognize items [35], application of active tDCS increases false alarm rates. These data further establish that measuring recognition memory using only a corrected measure (such as A′ or d′), as many prior tDCS studies have done, may gloss over important details of how tDCS may affect memory. In this study, reduced memory performance as measured by A′ was primarily driven by increased false alarms, not reduced hits. We recommend thoughtful interrogation of both hits and false alarms along with the corrected recognition measures in future work.

In this study we included a difficulty manipulation to further test the baseline hypothesis. Our reasoning for this was that memory support should be reduced for more difficult items than the easier items. Our difficulty manipulation did not interact with tDCS effects on memory performance, which somewhat mutes the baseline hypothesis interpretation. More research is needed to determine the degree to which individuals must over-perform or under-perform at baseline on certain tasks for tDCS to be beneficial. More repetitions may be necessary to increase baseline performance to a level at which older participants would show memory improvements under tDCS.

A few studies have shown beneficial effects of stimulation when baseline performance is low [49], so perhaps the true relationship between tDCS efficacy and baseline performance is nonlinear, or moderated by other variables related to participant age. For example, older adults show altered patterns of task-related brain activity [50], and electrode locations that facilitate performance in one population may not be evident in another. Indeed, past work has shown similar effects of tDCS in both younger and older adults, but only when stimulation was applied to different cortical regions for one group versus the other ([20, 51]; although see 17]. Thus, stimulating the same location in both younger and older adults may lead to different effects depending upon the population tested [52–54]. Clearly, the full relationship between tDCS effects and baseline performance requires further study.

Greater perception of sensations (e.g., itching) correlated negatively with performance on the recognition task. This effect was not present in our prior study with younger adults [31], so perhaps increased sensations disrupted successful memory encoding processes for our older adult participants to a greater degree than it did in the younger adults. Older adults begin with fewer processing resources to devote to cognitive tasks [55], so partial distraction may particularly affect them. This effect should be taken into account in future research in this area.

Examining ways to improve memory performance in older adults is an important and rich area of study [56–61]. As an intervention, tDCS shows promise in improving memory, but more work is necessary to understand the conditions under which tDCS might lead to improved memory performance in older adults. We found that tDCS increases false alarm rates when stimulation is applied over the left inferior frontal gyrus at 2.0mA, but stimulation to other scalp locations and/or at different current strengths might improve memory.

## Conclusions

Our results suggest that tDCS applied over the left inferior frontal gyrus increases false alarm rates during a face–name associative memory task, which could lead to decreased memory performance. In conjunction with prior results with younger adults, our current findings indicate that tDCS may be effective when baseline performance is high, but decreases performance when baseline is low. Future research is needed to identify the specific effects of tDCS on cognitive ability, and how this relationship is moderated by baseline cognitive performance.

## Figures and Tables

**Table 1:** Demographics/neuropsychological measures of study participants

	Condition	
Measure	Active *M* (*SD*)	Sham *M* (*SD*)
Females:Males	5:2	4:3
Age	72.86 (6.01)	70.57 (6.05)
Health rating^a^	4.00 (0.58)	4.14 (0.90)
Health satisfaction	3.71 (0.76)	4.14 (1.07)
Digit comparison	54.29 (11.13)	57.86 (10.95)
Digit symbol	28.57 (8.16)	31.00 (8.33)
Digit span	11.00 (3.65)	13.43 (6.16)
Verbal fluency	86.43 (18.28)	87.71 (20.11)
Vocabulary*	33.71 (2.14)	36.71 (1.38)
MMSE	27.29 (1.98)	28.00 (1.53)

* Difference between active and sham condition, significant at *p < 0.05*

a Health questionnaire ratings on scale of 1–5

Abbreviations: SD, standard deviation; MMSE, Mini Mental State Examination

**Table 2:** Behavioral means

	1 Trial		2 Trials	
	Active *M* (*SD*)	Sham *M* (*SD*)	Active *M* (*SD*)	Sham *M* (*SD*)
Recall	0.02 (0.02)	0.09 (0.09)	0.09 (0.07)	0.23 (0.20)
HR	0.80 (0.14)	0.84 (0.20)	0.89 (0.11)	0.91 (0.11)
FAR	0.79 (0.19)*	0.49 (0.21)	0.73 (0.15)*	0.48 (0.23)
A′	0.47 (0.22)*	0.76 (0.18)	0.68 (0.15)	0.81 (0.14)

* Difference between active and sham conditions, significant at *p < .05*

Abbreviations: SD, standard deviation; HR, hit rate; FAR, false alarm rate

**Table 3:** Sensation measures by time point

Measure	Itching		Burning		Tingling		Mental fatigue	
	Active	Sham	Active	Sham	Active	Sham	Active	Sham
Time point	*M* (*SD*)	*M* (*SD*)	*M* (*SD*)	*M* (*SD*)	*M* (*SD*)	*M* (*SD*)	*M* (*SD*)	*M* (*SD*)
*Time 0*	3.29 (1.70)*	1.14 (0.38)	2.86 (1.77)*	1.14 (0.38)	2.29 (1.80)	1.14 (0.38)	1.43 (0.79)	1.00 (0.00)
*Time 1*	2.57 (1.72)	1.29 (0.76)	2.71 (1.50)*	1.14 (0.38)	2.00 (1.53)	1.14 (0.38)	1.43 (0.79)	1.00 (0.00)
*Time 2*	2.43 (1.62)	1.14 (0.38)	2.57 (1.62)	1.29 (0.49)	2.00 (1.53)	1.29 (0.76)	1.43 (0.79)	1.00 (0.00)
*Time 3*	2.57 (1.99)	1.29 (0.76)	2.71 (1.80)	1.29 (0.49)	2.00 (1.53)	1.14 (0.38)	1.29 (0.49)	1.14 (0.38)
*Time 4*	2.86 (1.77)	1.29 (0.76)	2.43 (1.90)	1.29 (0.49)	2.14 (1.68)	1.14 (0.38)	1.43 (0.79)	1.14 (0.38)

Sensations were reported on a 1–10 scale

* Difference between active condition and sham condition, significant at *p* < 0.05.

Abbreviations: SD, standard deviation
